# Late onset hyperplastic callus formation in osteogenesis imperfecta type V simulating osteosarcoma—A case report

**DOI:** 10.1016/j.ijscr.2020.03.024

**Published:** 2020-03-28

**Authors:** Hans Christoph Vonderlind, Matthias Jessel, Alexander Knobel, Ingke Juergensen, Johannes Struewer

**Affiliations:** Department of Orthopaedics and Traumatology, University Hospital Oldenburg, Germany

**Keywords:** Osteogenesis imperfecta type V, Radiography, Hyperplastic callus formation, Simulation of osteosarcoma

## Abstract

**Introduction:**

We report a case of late onset hyperplastic callus formation (HPC) in the right femur in type V osteogenesis imperfecta (OI) mimicking the occurrence of a malignant osteosarcoma.

**Presentation of case:**

A 27-year-old female patient consulted us due to swelling in her right femur over 2–3 months without trauma. X-rays looked like an osteosarcoma, blood tests showed increased bone metabolism. A CT scan, a MRI and biopsy were performed, showing late onset HPC due to osteogenesis imperfecta type V.

**Discussion:**

OI shows a heterogeneous disease pattern due to a variety of clinical and radiographic findings. HPC is a rare complication of OI type V. Differential diagnosis range from cortical or periosteal osteosarcoma, periostitis, myositis ossificans, subperiosteal hematoma secondary to trauma or osteomyelitis.

**Conclusion:**

Recognition of HPC as a form of this particular type of OI is important to avoid misdiagnosis like malignant transformation to osteosarcoma. A biopsy and advanced imaging modalities like CT, MRI and scintigraphy are recommended.

## Highlights

• Hyperplastic callus formation is a very rare complication of osteogenesis imperfecta type V.
• The most important differential diagnosis is malignant osteosarcoma.
• A biopsy is the only way to distinguish between those two conditions.
• The underlying pathophysiology is still unknown and there is no causal therapy.
• A multidisciplinary approach in diagnosis and therapy is mandatory.

## 1 Introduction

This case report is in accordance with the SCARE guidelines [1]. Osteogenisis imperfecta (OI) represents a heritable disorder which is among other things characterized by brittle bones, deformity of the spine and long bones. Based on clinical and radiographic findings Sillence firstly classified OI in four types I–IV [2]. Types V and VI were added by Glorieux et al., type VIII added by Cabral et al. [3,4]. Skeletal manifestations are based on a generalized deficiency of development of both membranous and endochondral bone including osteopenia, multiple fractures and wormian bone formation [5]. In OI types I–IV, the disease commonly is caused by mutation in the two genes encoding for the collagen type I alpha chains [6]. These mutations are absent in OI types V–VII. OI type V is characterized radiologically by interosseous membrane calcification of the forearms and a radiodense band visualized at the growth plate. The clinically and radiologically most conspicuous feature and rare complication of OI type V is the formation of HPC [4,[7], [8], [9], [10], [11], [12]]. The main challenge is the differentiation of HPC to malignant osteosarcoma [13].

## 2 Case report

A 27-year-old female patient presented with an indolent but progressive swelling of the right mid femur over the last 2–3 months in our outpatient consultation hour of our university hospital. She reported of a known osteogenisis imperfecta type V, being the only member of her family suffering from it. Clinical phenotype was characterized by short statue, a mild kypho-scoliosis, a short neck and a mild ligamentous hyperlaxity. She did not report of a trauma or any inflammatory disease. Although numerous fractures of the lower extremities occurred over the last decade which were treated conservatively as well as operatively by intramedullary nailing, HPC or extraordinary swelling never developed.

**Fig. 1:**
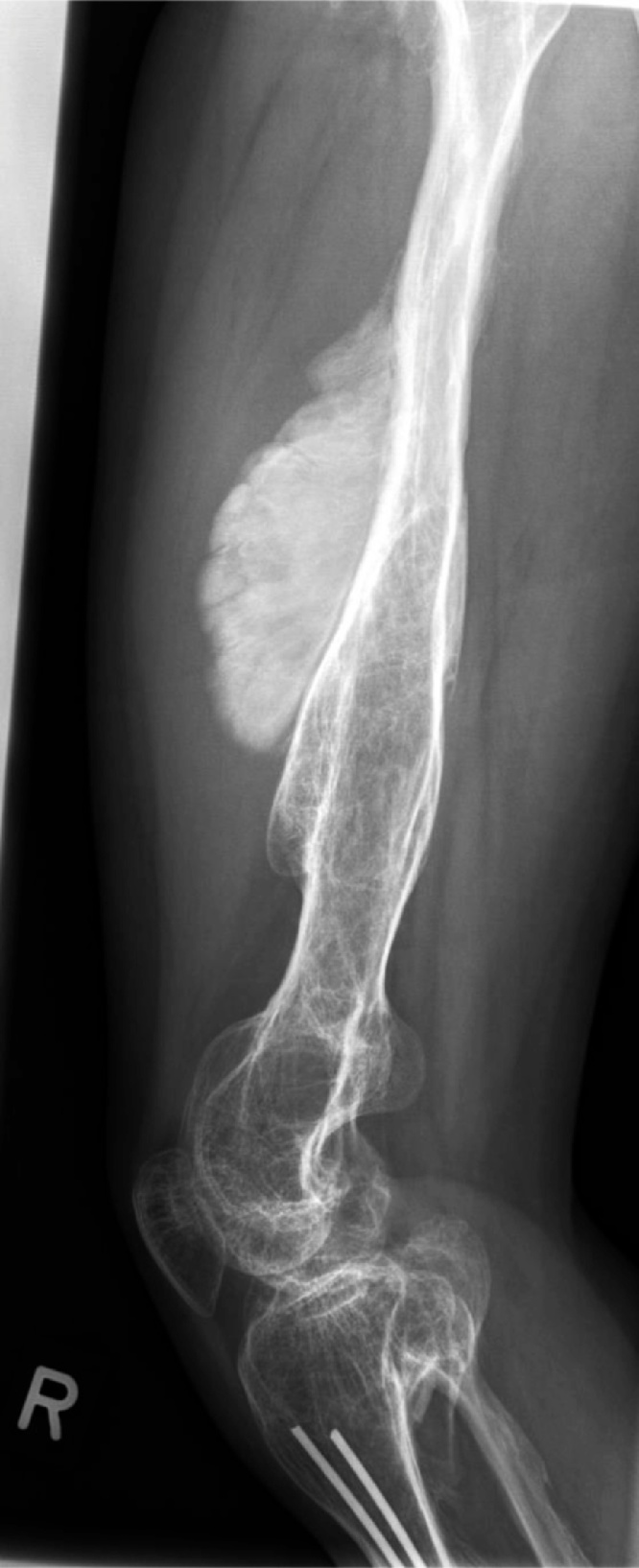
Radiographic imaging of the femoral shaft in anterior-posterior and lateral views showing a massive irregular periosteal hypertrophic lamellar ossification at with an irregular sunburst pattern as well as the appearance of spiculae. Furthermore the radiographs show the condition of intramedullary nailing for a tibial shaft fracture years ago.

Conventional radiographs of the affected area showed a massive osseous tumor of the right femoral mid-shaft (Fig. 1). Radiographically the lesion was characterized by a massive irregular hypertrophic lamellar ossification with an irregular sunburst pattern as well as the appearance of spiculae, showing potential periosteal signs of malignancy. Laboratory tests only showed an elevated level of alkaline phosphatase of 257 U/l.

**Fig. 2:**
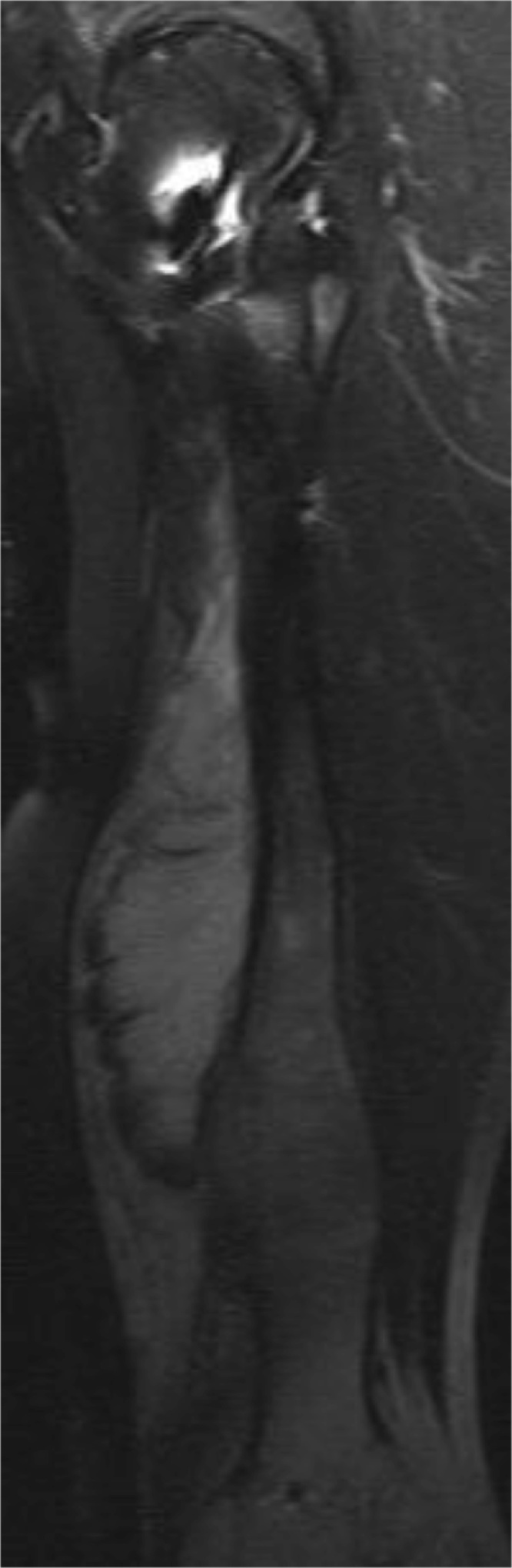
Magnetic resonance Imaging of the right femur in anterior-posterior view showing a massive irregular periosteal hypertrophic lamellar ossification at with an irregular sunray pattern. An additional infiltration of the surrounding musculature and soft tissue cannot be clearly ruled out.

**Fig. 3:**
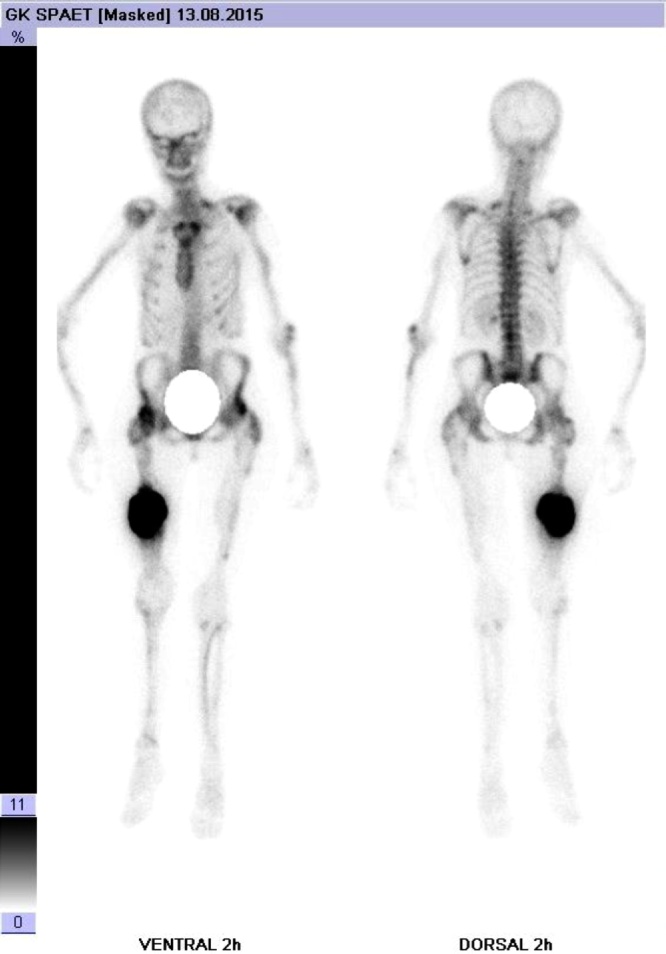
Skeletal scintigraphy showing a massive enhanced accumulation at the mid femoral shaft and furthermore the scintigraphy gives no evidence of metastatic lesion.

In the assumption of a malignant osteosarcoma, further tumor staging was initiated, including contrasted computed tomography of thorax, abdomen and pelvis. Furthermore a contrasted magnetic resonance Imaging (MRI) (Fig. 2) of the affected femur and a scintigraphy (Fig. 3) were performed. MRI showed massive irregular periosteal hypertrophic lamellar ossification with an irregular sunray pattern, additional infiltration of the surrounding musculature and soft tissue could not be clearly ruled out. Skeletal scintigraphy showed a massive enhanced tracer accumulation in the mid femoral shaft, but, as well as contrasted computed tomography of thorax, abdomen and pelvis showed no additional lesions. A mini-open biopsy under consideration of tumor-surgical aspects was performed by two senior surgeons. Histopathologic analysis of the bioptic specimen showed distinctive zones with the outer regions of callus containing edematous tissue with a loose collagenous network to the innermost region showing hypercellular trabeculae of woven bone and small cartilaginous islands without signs of malignant transformation. In consideration of all findings, the diagnosis of late onset HPC in a patient with osteogenesis imperfecta type V was made and the differential diagnosis of a cortical or periosteal osteosarcoma was ruled out. The patient wished no additional surgical intervention like marginal resection of the HPC and preferred to wait for possible spontaneous remodeling [9,14]. She undergoes regularly 6-month follow-up examinations.

## 3 Discussion

OI remains a heterogeneous disease pattern. Diagnosis might be challenging and includes a variety of clinical and radiographic findings [4,6].

HPC is a rare complication of osteogenesis imperfecta type V and its occurrence has only been reported in about 30 cases, especially in adult patients it remains a very rare appearance [6,11]. Most often HPC formation was observed in children or adolescents. The occurrence of HPC might include variable skeletal sites. The lower extremities are most commonly involved with predominance of the femora, but HPC may also affect the tibia, the humerus and the forearm bones [4,9,10,14]. HPC formation might arise in the presence or absence of prior known fractures or seen following surgical interventions as an isolated or repeated event [9,11,13,14]. As mentioned, the clinical picture is variable and might consist of painful or painless swelling and enlargement of the extremity and low-grade fever over the course of weeks or month. Especially in younger patients, both, pain and swelling, can remain steady or decrease over time. Moreover patients report of warmth and tenderness over the affected area and the overlying skin might become taut [6,[9], [10], [11],^13^,^14^]. Analysis of laboratory values might show an elevation of the sedimentation rate and an increased level of alkaline phosphatase, both reflecting to the elevated bone metabolism. Leukocytosis or elevation of further laboratory values with regard to inflammation have not been reported [4,9,13].

Diagnosis of HPC formation is challenging and varies with patients age. During childhood the differential diagnosis are idiopathic osteoporosis, periosteal tumors, bleeding disorders, juxtacortical myositis ossificans, osteomyelitis or even child abuse injury and domestic violence [10,11,[14], [15], [16]]. However, periosteal reaction in battered child syndrome shows different stages of evolution, extraosseous soft tissues lesions are usually present and wormian bones are absent. Therefore analysis of the patient`s medical history correlated with phenotype is very important.

In adults the diagnosis of HPC formation in OI Type V may be more difficult because of superimposed traumatic and osteoarthritic changes. Differential diagnosis includes amongst others cortical or periosteal osteosarcoma, periostitis, myositis ossificans, subperiosteal hematoma secondary to trauma or osteomyelitis [10,11,[13], [14], [15], [16]]. Main focus with regard to literature remains to differentiate benign HPV formation from malignant osteosarcoma [6,9,15,16].

The clinical picture with pain and swelling is similar and elevation of alkaline phosphatase may be seen in both. The radiologic features with new bone formation without well-defined edges are very similar in both entities, although some features may be helpful in distinguishing between these two conditions [12]. In osteosarcoma the cortical bone is eroded whereas it normally remains intact in HPC, although this feature is inconstant and not always visible. Further radiologic patterns include dense, almost lobulated callus without any crossing of joint lines. In later stages the HPC diminishes in bone density and gets a nearly halisteretic appearance [15,16].

An osteosarcoma shows a tendency towards a focal appearance, reflecting the concentric growth of the tumor, while HPC usually grows along the shaft axis of the bone. The characteristic but infrequent sunray pattern of osteosarcoma may look like the early stages of HPC. In accordance with other authors we emphasize the use of advanced imaging modalities like CT, MRI and scintigraphy besides complete skeletal survey and x-ray imaging [6,9,12,14,16]. Complete tumor staging including contrasted computed tomography of thorax, abdomen and pelvis as well as contrasted MRI of the affected regions are of vital importance. Given the rarity of this disorder, multicenter collaborations are advisable [6,9,[12], [13], [14],^16^]. The final distinct differentiation between osteosarcoma and HPC cannot be made on clinical and radiologic features, even under use of advanced imaging modalities, therefore a biopsy is obligatory to distinguish between those two conditions [[9], [10], [11],^15^,^16^]. The typical histological appearance on bone biopsy with a mesh-like lamellation without malignant cell transformation is not seen in other types of OI and differentiates HPC from osteosarcoma. Further histopathologic features of rapidly growing HPC consist of distinctive zones with the outer regions of callus containing edematous tissue with a loose collagenous network to the innermost region showing hypercellular trabeculae of woven bone and small cartilaginous islands [4,6,13]. The distinct pathophysiology of HPC formation is still unclear. Several lines of evidence are compatible with the hypothesis that dysregulated periosteal osteogenesis is one of the factors involved [13,17]. Cho et al. encountered a single recurrent mutation in the 5-untranslated region of IFTM5 encoding interferon-induced transmembrane protein 5 [17]. At present, there is no evidence that HPC formation can be influenced by medical treatment approaches.

## 4 Conclusion

HPC formation is a potentially serious complication of OI type V, occurring predominantly in the long bones of OI type V patients. Lesions can be precipitated by fractures, arise spontaneously and can become very large, altering the architecture of the bone. Recognition of HPC is important in order to avoid misdiagnosis like malignant transformation to osteosarcoma and differential diagnosis is only possible by biopsy.

## Declaration of Competing Interest

None of the authors has a conflict of interest.

## Funding

This research did not receive any specific grant from funding agencies in the public, commercial, or not-for-profit sectors.

## Ethical approval

Since this is a case report, no ethical approval had to be obtained by our institutional review board.

## Consent

Informed patient consent for publication of this case report was obtained.

## Registration of research studies

This case report is not part of a clinical study.

## Guarantor

Johannes Struewer, MD

Department of Orthopedics and Traumatology, University Hospital Oldenburg, Rahel-Straus-Straße 10, 26133 Oldenburg, Germany.

## Institutional review board

Since this is a case report, no ethical approval had to be obtained by our institutional review board.

## Provenance and peer review

Editorially reviewed, not externally peer-reviewed.

## CRediT authorship contribution statement

**Hans Christoph Vonderlind:** Writing - original draft, Investigation. **Matthias Jessel:** Writing - review & editing. **Alexander Knobel:** Investigation. **Ingke Juergensen:** Conceptualization. **Johannes Struewer:** Supervision, Writing - review & editing.
